# Exogenous Abscisic Acid Mediates Berry Quality Improvement by Altered Endogenous Plant Hormones Level in “Ruiduhongyu” Grapevine

**DOI:** 10.3389/fpls.2021.739964

**Published:** 2021-10-01

**Authors:** Jiajia Li, Boyang Liu, Xiangyi Li, Dongmei Li, Jiayu Han, Ying Zhang, Chao Ma, Wenping Xu, Lei Wang, Songtao Jiu, Caixi Zhang, Shiping Wang

**Affiliations:** ^1^Department of Plant Science, School of Agriculture and Biology, Shanghai Jiao Tong University, Shanghai, China; ^2^Grape and Wine Institute, Guangxi Academy of Agricultural Sciences, Nanning, China

**Keywords:** ABA, phytohormones, fruit quality, correlation analysis, grape

## Abstract

Abscisic acid (ABA) plays a key role in fruit development and ripening in non-climacteric fruit. A variety of metabolites such as sugars, anthocyanins, fatty acids, and several antioxidants, which are regulated by various phytohormones, are important components of fruit quality in grape. Here, grape cultivar “*Ruiduhongyu*” was used to investigate the relationship between endogenous phytohormones and metabolites associated to grape berry quality under exogenous ABA treatment. 500 mg/L ABA significantly improved the appearance parameters and the content of many metabolites including sugar, anthocyanin, and other compounds. Exogenous ABA also increased the contents of ABA, auxin (IAA), and cytokinins (CTKs), and transcription level of ABA biosynthesis and signaling related genes in fruit. Furthermore, a series of genes involved in biosynthesis and the metabolite pathway of sugars, anthocyanins, and fatty acids were shown to be significantly up-regulated under 500 mg/L ABA treatment. In addition, Pearson correlation analysis demonstrated that there existed relatively strong cooperativities in the ABA/kinetin (KT)-appearance parameters, ABA/IAA/KT-sugars, ABA/indolepopionic acid (IPA)/zeatin riboside (ZR)-anthocyanins, and gibberellin 3 (GA_3_)/methyl jasmonate (MeJA)-fatty acids, indicating that 13 kinds of endogenous phytohormones induced by ABA had different contributions to the accumulation of quality-related metabolites, while all of them were involved in regulating the overall improvement of grape fruit quality. These results laid a primary foundation for better understanding that exogenous ABA improves fruit quality by mediating the endogenous phytohormones level in grape.

## Introduction

Grape (*Vitis vinifera* L.), one of the most important and oldest cultivated fruit crops, is originated from western Asia (El-Mashharawi et al., 2020). Due to the versatility of its fruit products (fresh eating and wine making) and good economic benefits, grape plantations are popular among orchardists and widely distributed around world (Reisch et al., 2012). The fruit quality is the fundamental factor determining processing method (juice and raisin making) and commodity value. Appearance quality including fruit shape, firmness, and coloring are an important part of fruit quality, in which anthocyanins are the main pigment substances determining the coloring of table grapes and wines (De Orduna, 2010). A variety of metabolites are involved in the quality formation of grape fruit, in which sugars, organic acid, amino acids, and fatty acids (FAs) play a vital role in fruit flavor (Lado et al., 2018). Moreover, there are also a variety of nutrients in grape, such as flavonoids, L-ascorbic acid, polyphenol, and resveratrol, which could alleviate neuronal and cardiovascular disease due to their antioxidant activity (Liu et al., 2018; Shiraishi et al., 2018). It has been widely acknowledged that molecular breeding is an effective way to obtain new species with good fruit traits in woody perennial fruit crops (Töpfer et al., 2011). However, considering the contradictoriness between the long breeding cycle and urgent market demands, fast and effective improvement methods are a priority to researchers. In recent decades, many studies had demonstrated that using exogenous harmless substances could be a feasible strategy to significantly improve fruit quality, including exogenous plant hormones, plant growth regulators, amino acids, and antioxidants (Cai et al., 2014; Raza et al., 2014). For example, 400 mg/L exogenous ABA sprayed on grapevine at the veraison stage could promote the coloration of pericarp (Shahab et al., 2019), and 400 and 600 mg/L ABA treatment on grapevine significantly contributed to grape yield promotion (Mohamed et al., 2019).

Abscisic acid (ABA), as a vital plant hormone and signal molecule, plays a key role in reproductive organ development, stress response, and fruit ripening (Leng et al., 2018; Kou et al., 2021a). Its biosynthesis and signaling mechanism have been well studied in model plants. ABA biosynthesis generally has two pathways: C15 direct pathway and C40 indirect pathway. The former directly forms ABA through C15 farine pyrophosphate (FPP), the latter forms ABA indirectly through oxidative cleavage of carotenoids, which is the main pathway of ABA biosynthesis in higher plants (Seo and Koshiba, 2002). ABA signal transduction mainly contains a complex network of PYR/PYL/RCAR, ABA receptors, Clade A PP2Cs, and SnRK2s (Santiago et al., 2012). ASR peptide is confirmed to respond to ABA signal and plays an important role in regulating ABA signal transmission (Carrari et al., 2004). Many investigations have focused on the effects of ABA on the fruit development and ripening, especially in non-climacteric fruit species. It had been demonstrated that exogenous ABA treatment significantly enhanced the accumulation of sugars (Alferez et al., 2021), double NCED isozymes controlled ABA biosynthesis for ripening and senescent regulation in peach fruits (Wang et al., 2021). Furthermore, in climacteric fruit species, it had been suggested that overexpression of the abscisic acid β−glucosidase gene (*DkBG1*) altered fruit ripening in transgenic tomato (Liang et al., 2020). There were also some reports on the improvement of ABA on fruit quality and nutrient value in grapes, exogenous ABA spraying on grapevine contributed to anthocyanins, flavonoids, resveratrol, and other phenolics accumulation (Deis et al., 2011; Ju et al., 2016). Sugar accumulation determines the market value of table grapes (Liu et al., 2007), it had been reported that exogenous ABA could improve grape frost-resistance via increasing sugar content (Li et al., 2017), and ABA receptor VvSnRK1.1/VvSnRK1.2 interacting with WRKY22 transcription factor jointly regulated the accumulation of sugars in grapevine (Huang et al., 2021).

Plant hormones do not act in isolation in the process of plant growth, development, maturity, and senescence. On the contrary, they co-regulate a series of plant life activities in the complex network of phytohormones interaction (Fenn and Giovannoni, 2021; Li et al., 2021). Thus, to explore the crosstalk among endogenous plant hormones regulating fruit quality improvement seems to be meaningful. Previous study demonstrated that ABA and ethylene (ETH) co-regulated tomato ripening (Kou et al., 2021b), IAA and GA contributed to citrus fruit set and development (Bermejo et al., 2018). IAA and ETH interacted together to promote the production of carotenoid in tomato at the maturity stage (Cruz et al., 2018). ABA, JA, SA, and ETH jointly regulated abiotic stress defense response of apple fruits under photooxidation and heat stress (Torres et al., 2017). It had also been demonstrated that crosstalk between ABA and GA was regulated by ETH and sugar signals, which jointly regulated the increase of non-climacteric fruit sugars content (Alferez et al., 2021), in addition, IAA and ABA co-regulated sugar accumulation in pear at the late ripening stage (Lindo-García et al., 2020). Otherwise, interaction between ABA, ETH, and JA mediated the enrichment of anthocyanins in *Lycium* plants (Li G. et al., 2019). As previous studies stated, crosstalk among endogenous phytohormones could interact together to regulate fruit development and ripening, stress resistance enhancement, sugars, and anthocyanins accumulation, ultimately resulting in the prominent improvement of fruit quality.

The new table grape cultivar “*Ruiduhongyu*,” with various advantageous traits containing high-yield, precocity, pink coloring, and muscat flavor is selected from the bud mutation of “*Ruiduxiangyu*” (*Jingxiu × Xiangfei*), becoming a new variety with potential development in China (Xu et al., 2014). While in southern China, such as Guangxi province, it is difficult to obtain grapes with brilliant color in the ripening stage due to high temperature. Meanwhile, as a new table grape variety, studies on the effect of exogenous ABA on “*Ruiduhongyu*” fruit quality are still lacking. In this study, the effect of exogenous ABA on berry enlargement, coloration, and interior quality were discussed, whether exogenous ABA mediated a series of biosyntheses of endogenous phytohormones to regulate grape berry size, pericarp coloration, sugars accumulation, flavor formation, and stress resistance improvement were investigated, and the potential correlation between endogenous phytohormones variation and fruit quality-related metabolites was cleared out. The purpose of this study was to provide references for improving fruit quality via cultivation techniques amelioration.

## Materials and Methods

### Plant Material and Experiment Design

This research was established in a greenhouse in Grape and Wine Institute, Guangxi Academy of Agricultural Sciences (107°45*′* E, 22°13*′* N, NanNing, Guangxi, China) in 2020, using 5 years secondary *“Ruiduhongyu”* grape, a red bud mutation origin from *“Ruiduxiangyu” (Jingxiu × Xiangfei)* grape. Grapevines in both control groups and treatment groups were used as shelter cultivation with the same water and fertilizer condition supplied. Three whole grapevines were sprayed with exogenous ABA (ABA concentration: 500 mg/L; Spraying volume: 2L) at the veraison stage on November 20th (DAA 35). Thirty grape berries of a uniform size were collected from each 500 mg/L ABA treatment group and the control group at 5 specific sampling dates: DAA 40, November 25th; DAA 45, November 30th; DAA 53, December 8th; DAA 69, December 24th; DAA 75, December 30th, DAA was indicated as days after anthesis. All samples were immediately mailed back to the laboratory in Shanghai Jiao Tong University (121°29*′* W, 31°11*′* N, Shanghai, China) and stored in a −80°C refrigerator for further experiments. After physiological parameters had been measured, grape berries were grinded in liquid nitrogen and then the powder was stored in a −80°C refrigerator, all tests were implemented for at least three biological duplications.

### Physiological Parameters Analysis

Thirty berries in each sampling stage without visible damage were selected for berry weight, longitudinal and transverse diameters, total soluble solid (TSS) and titratable acid (TA) measurements. Berry weights were measured by analytical balance (Sartorius, German), longitudinal and transverse diameters were quantified by vernier caliper (Mitutoyo, TKY, Japan), TSS was measured by refractometer (OWELL, Hangchow, CHN) using 1 mL of grounded and fully squeezed grape juice, and represented as °Brix (Sánchez-Moreno et al., 2003). TA was measured by potentiometric titrator (HAINENG, SZ, CHN) and represented as g/L (Sánchez-Moreno et al., 2003). All tests were implemented for at least three technical duplicates.

### Quality-Related Parameters Quantification

According to the previous methods, reducing sugar was measured using 3, 5-dinitrosalicylic acid method with a little adjustment (Bonilla et al., 1999). The specific extraction process was as follows, firstly, 1 g of accurately weighed freeze-dried powder and 25 mL of deionized water were added in a centrifuge tube. Then the solution continued reacting for 30 min in a constant temperature water bath (JingHong, SHH, CHIN) at 80°C. After two filtrations, the liquid volume was metered to 100 mL by deionized water and then fully mixed. Finally, 2 mL of reducing sugar extracting solution and 1.5 mL of 3, 5-dinitrosalicylic acid reagent were fully blending, then the absorbance value in each sample was analyzed by spectrophotometer under 540 nm wavelength and converted to “glucose equivalents” according to the calibration curve prepared with a d-(+)-glucose.

The quantification of soluble sugar was referred to the anthrone colorimetry assay with a little adjustment (Bonilla et al., 1999), 50 mg freeze-dried grape powder and 4 mL 80% ethanol was added in a 10 mL graduated centrifuge tube. Then, the sample was placed in a water bath at 80°C for 40 min, after centrifugating at 4,000 rpm for 10 min, the supernatant was collected. Next, the residue was added with 2 mL 80% ethanol and centrifugated at 4,000 rpm for 10 min again. Supernatant was incorporated and 10 mg of activated carbon was added, decolorized at 80°C for 30 min, then 80% ethanol was added and constant solution volume to 10 mL. One milliliter of filtrate was added with 5 mL of anthrone reagent, then fully mixed and boiled in a boiling water bath for 10 min. After the solution was cooled to the room temperature, its absorbance value was measured at 625 nm by spectrophotometer.

The extraction of polyphenol and soluble dietary fiber method was as follows (Gorinstein et al., 1999). Firstly, 1 g of dry sample was accurately weighed, then 80% methanol and 1% hydrochloric acid (solid-liquid ratio = 1:30) were added. Next, under ultrasonic extraction reacting at 45°C for 40 min, the supernatant was collected for polyphenol, anthocyanins, and flavonoid quantifications, and residue was collected for dietary fiber determination. Soluble dietary fiber was measured as follows, 0.4 M hydrochloric acid (solid-liquid ratio = 1:15) was added to the residue, after vigorously shaking, the solution was dried in 80°C oven to constant weight, dry weight was measured by analytical balance (Sartorius, German) and reported as g. Polyphenol was measured as follows, 3 mL of 0.2 M Folin-Ciocalteau reagent was added to 3 mL of 10 times diluted supernatant, then 2.4 mL of 0.7 M Sodium carbonate solution was added. After mixture had been reacting for 120 min under no light condition, its absorbance was determined under 760 nm wavelength. The content of polyphenol in each sample was calculated as gallic acid equivalents (GAE) and reported as mg/g.

The method for determination of flavonoid content was implemented by the vanillin colorimetric assay with the calibration standard of (+)-catechin hydrate (Price et al., 1978). Firstly, 0.2 mL of supernatant extracting solution and 60 μL of 5% sodium nitrite solution were mixed, after fully blending and reacting for 6 min, then 0.8 mL of 1 M sodium hydroxide solution and 3.88 mL of 50% ethanol solution were added in turn. Finally, after mixture had been standing for 15 min, its absorbance was determined under 510 nm wavelength. The flavonoid content was calculated as rutin equivalent per gram of sample.

Total anthocyanin content was determined by PH differential method (Porter et al., 1985), 2.5 mL of supernatant extracting solution was added to reach a constant volume of 10 mL using 0.025 M potassium chloride buffer (PH = 1.0) and 0.4 M sodium acetate buffer (PH = 4.5), then the absorbance of two kinds of solution were determined under 520 nm wavelength and 700 nm wavelength. The total anthocyanin content in each sample was reported as mg/g.

Soluble protein was measured according to Coomassie Bright Blue assay (Singleton and Rossi, 1965). 2.0 g of freeze-dried sample and 5 mL of deionized water were added to a centrifuge tube, then the homogenate was centrifuged under 12,000 rpm for 15 min at 4°C. Finally, 1 mL of collected supernatant and 5 mL of Coomassie Bright Blue solution were fully mixed. Finally, the mixture was determined the absorbance under 595 nm wavelength, the concentration of soluble protein in each sample was reported as mg/g.

The method for determination of L-ascorbic acid content was as follows (Bonilla et al., 1999), Firstly, 1 g of dry sample was taken and 2 mL of 50 g/L TCA solution was added, after sufficiently dissolving, the volume was adjusted to 10 mL using 50 g/L TCA solution again. Finally, 1 mL of extracting solution was added to 1 mL of 50 g/L TCA solution, after fully blending, the solution absorbance was determined under 534 nm wavelength. The ascorbic acid content was calculated as ascorbic acid equivalent per gram of sample.

The extraction of resveratrol was as follows: 3 g of freeze-dried powder and 10 mL of anhydrous ethanol were added in a centrifuge tube, then placed under ultrasonic treatment for more than 30 min. The obtained liquor was filtered by 0.22 μm membrane and its absorbance was determined under 525 nm by spectrophotometer.

The methods for determination of malondialdehyde (MDA), proline (Pro), guaiacol peroxidase (POD), superoxide dismutase (SOD), catalase (CAT), ascorbate peroxidase (APX), glutathione (GSH), and DPPH free radical scavenging rate were referred to Nahakpam and Shah (2011) with a little adjustment, all tests were implemented with at least three technical duplicates. MDA measurement was as follows: 0.5 g of freshy fruit was taken, 2 mL of 10% TCA solution was added for grinding, and then 5 mL of TBA solution was added for mixing. After reacting in a boiling water bath for 10 min, the liquid was centrifuged at 3,000 rpm for 10 min, then solution absorbance was, respectively, measured at 532, 600, and 450 nm, and MDA content was reported as μmol/g. Pro measurement was as follows, 0.5 g of flesh tissue was weighed, after reacting in a boiling water bath for 10 min, 2 mL of glacial acetic acid and 2 mL of acidic hydrin were added. Next, after reacting in a boiling water bath for 30 min, 4 mL of toluene was added after cooling. After centrifugation (3,000 rpm, 5 min), the supernatant absorbance was measured at 520 nm and final concentration was reported as μg/g. POD, SOD, and CAT measurements were as follows: Firstly, 0.5 g of flesh fruit was weighed, a small amount of phosphoric acid (PBS) buffer (0.05 mol/L, PH = 7.8) was added and ground into homogenate, then the volume was kept to a constant of 9 mL with PBS buffer. After centrifugation (4°C at 3,000 rpm) for 10 min, the supernatant was stored at 4°C for further experiments. For POD detection, 3 mL of enzyme solution was added 56 μL of guaiacol, heated until the color faded, then cooled to room temperature. After adding 38 μL of 30% hydrogen peroxide, the POD activity was determined at 470 nm. For SOD detection, 2.5 mL of the reaction mixture (contained 130 mmol/L of Met, 750 μmol/L of NBT, and 100 μmol/L of EDTA-Na_2_) was added 0.2 mL of 0.05 M phosphate buffer (pH = 7.8) in a tube, 0.2 mL of enzyme solution was added to another tube, and 0.3 mL of 20 μmol/L riboflavin solution was quickly added to each tube. After 15 min reaction, the absorbance was measured at 560 nm and SOD activity was reported as mmol/g FW. CAT detection was measured as follows: 0.2 mL of enzyme solution was added to 1.5 mL of 0.2 mol/L PBS buffer (pH 7.8), 1 mL of distilled water and 0.3 mL of 0.1 mol/L hydrogen peroxide were added, then absorbance was detected at 240 nm and CAT activity was reported as U/g FW/min. For APX detection, 1.8 mL of 50 mmol/L phosphate buffer (pH 7.0), 0.1 mL of 15 mmol/L AsA, 0.1 mL of enzyme solution, and 1 mL of 0.3 mmol/L H_2_O_2_ were added to the test tube. Then, the change of OD_290_ was measured within 3 min with no enzyme solution (instead of PBS solution) as blank, the APX activity was reported as U/g FW. For GSH detection, 0.5 g of fruit was weighed and 5 mL of 5% trichloroacetic acid was added. After centrifugation at 15,000 rpm for 10 min, the absorbance of the supernatant was determined at 412 nm, and the final content was reported as μmol/g. DPPH free radical scavenging rate quantification was as follows: 4 mL of enzyme solution and 4 mL of DPPH solution were fully mixed. Then, it was brought to a constant volume of 10 mL using anhydrous ethanol. After full mixing, the solution absorbance was measured at 517 nm and DPPH free radical scavenging rate was reported as %.

### Endogenous Phytohormones Quantification

Endogenous phytohormones were extracted by known method with a little adjustment (Kojima et al., 2009), and detected by HPLC. The detection and quantification limits of target compounds by HPLC were shown in Supplementary Table 2. Firstly, 100 mg of freeze-dried powder was dissolved in the 1 mL of extracting solution (Methanol: Formic acid: ddH_2_O = 15:1:4) and stored in −20°C refrigerator for one night. The solution was then centrifugated (4°C, 13,000 rpm/min) for 20 min twice, incorporated the supernatant, and filtered by CNWBOND HC-C18 SPE Cartridge (CNW, German). Then, after rotary evaporating (YARONG, SHH, CHN) at 42°C residue was redissolved in 5 mL of 1 M formic acid. The liquid was immediately transferred into Poly-Sery MCX SPE Cartridge (CNW, German), first eluted by 5 mL of 1 M formic acid then eluted by 5 mL of methanol. Finally, after rotary evaporating again at 42°C, residue was redissolved using 0.5 mL of extraction solution (Methanol: Isopropanol: Acetic acid = 20:79:1), and the liquid containing abscisic acid (ABA), auxin (IAA), indolebutyric acid (IBA), indolepopionic acid (IPA), salicylic acid (SA), gibberellin_3_ (GA_3_), jasmonic acid (JA), and methyl jasmonate (MeJA) was acquired. Next, 5 mL of 0.35 M ammonia and 5 mL of 0.35 M ammonia in 60% methanol were immediately added into the same Poly-Sery MCX SPE Cartridge (CNW, German) in turn, then after the filter liquor was evaporated at 60°C, the residue was added 0.5 mL of 5% acetonitrile. Liquid containing zeatin (ZT), zeatin riboside (ZR), N-6 isopentenyl adenine (ip), N-6 isopentenyl adenine nucleoside (ipR), and kinetin (KT) was obtained.

The detection of endogenous phytohormones was performed on a LC3000 Semi-preparation Isocratic HPLC System (CXTH, BJ, CHN). A UV-detector (CXTH, BJ, CHN) and Capecell PAK C18 column (4.6 mm × 100 mm, 1.8 μm) was installed, the flow rate was adjusted to 0.8 mL/min. The elution system was first set as follows: 0–4 min, 20% A, 80% B; 4–8 min, 50% A, 50% B; 8–20 min, 80% A, 20% B; 20–22 min, 80% A, 20% B; 22–22.2 min, 20% A, 80% B. Under this program, ABA, IAA, IBA, IPA, SA, GA_3_, JA, and MeJA were, respectively, eluted in different retention times. Then, mobile phase A (0.06% acetic acid water) and mobile phase B (0.06% acetic acid-methanol) was changed, injection volume was still 20 μ, while flow rate was adjusted to 0.5 mL/min. The gradient program was set as follows: 0–8 min, 99% A-55% A; 8–14 min, 55% A-30% A; 14–16 min, 30% A-1% A; 16–24 min, 1% A-99% A. Under this program, ZT, ZR, ip, ipR, and KT were eluted in different retention times. UV wavelength was 254 nm and the temperature of the chromatographic column was 40°C. All reagents of chromatography grade were purchase in ANPEL (SHH, CHN), liquid for HPLC detection was filtered through a 0.22 μm membrane.

### Fatty Acids Measurement

The extraction of different FAs was implemented according to the following method (Song et al., 2019), powder stored in −80°C refrigerator was transferred to 4°C refrigerator to thaw, then 3 mL of grape juice and 3 mL of n-hexane were fully mixed. After the mixture was placed in the shaking table (TENSUC, SHH, CHN) under 300 rpm for 15 min, liquid supernatant was collected and methyl dodecanoate (0.4 mg/mL) was added immediately. After nitrogen blew (Organomation, United States) to 1 mL, the liquid was immediately methylated by known method. Firstly, the solution was added to 3 mL of 2% sodium hydroxide-methanol, then placed in a water bath at 85°C for 30 min. Next, 3 mL of 14% boron trifluoride-methanol solution was added, then placed in a water bath at 85°C for 30 min again. After the solution was cooled to room temperature, 1 mL of n-hexane was added and then shocked for 2 min, and left to stand for 1 h. Finally, 100 μ of supernatant was collected and brought to a constant volume of 1 mL using n-hexane, after extraction solution was filtered by 0.22 μm membrane and then detected in GC.

Then, J&W DB-WAX capillary column (30 mm × 0.25 mm, 0.25 μm) was installed in the GC system, inlet temperature was set to 250°C and the fluid intake was 1.0 μL, the split ratio was set to 20:1 and carrier gas was high-purity helium with a flow rate of 1.0 mL/min. The initial temperature was set to 180°C for 5 min, and then increased to 230°C at a rate of 3°C/min till the set temperature was reached.

### Fructose, Sucrose, and Glucose Measurements

The extraction of fructose, sucrose, and glucose contents referred to the known method (Nowicka et al., 2019), and was detected by HPLC. Firstly, 3 mL of grape juice was centrifuged under 10,000 rpm/min for 15 min. Then in a centrifuge tube, 1 mL of supernatant and 9 mL of ultrapure water was added. Finally, mixture liquid was filtrated through 0.22 μm membrane and detected by HPLC.

The standard curve was drawn as follows: firstly, glucose, sucrose, and fructose standard products were diluted with ultra-pure water into 100 ng/mL mixed liquid as the mother liquor, then the mother liquor was diluted in a gradient. Finally, the obtained solution was detected by HPLC, the standard curves of glucose and fructose were drawn, respectively, with the sugar content as the abscissa and the peak area as the ordinate. The determination method of each sample was consistent with the standard liquid measurement and repeatedly measured 3 times, then the corresponding sugar content was converted according to the standard curve, and the mean value as well as the standard deviation was calculated.

Detection of sugar levels was performed on LC3000 Semi-preparation Isocratic HPLC System (CXTH, BJ, CHN), differential detector (KNAUER, GERMAN), and Amino column (250 mm × 4.6 mm, 5 μm) was installed, mobile phase was acetonitrile-water (75:25, with a small amount of ammonium hydroxide). The flow rate was 0.6 mL/min and the column temperature was 25°C.

### Real-Time RNA-Seq Analysis

The extraction of total RNA was performed on RNA prep Pure Plant Plus Kit (TaKaRa, Dalian, China) using berries collected in all five sampling stages from both 500 mg/L ABA treatment group and the control group. The purity and integrality analyses were established on BIO-RADXR gel imaging analysis system (Bio-Rad, CA, United States). Referencing to the manufacturer’s instructions, the PrimeScript^TM^ RT reagent kit with gDNA Eraser (Perfect Real Time) (TaKaRa, Dalian, China) used 1 μg of total RNA extracted from berries, aiming to obtain the first strand of cDNA.

10 μL of final volume consisted of 1 μL of cDNA, 5 μL of TB Green^®^ Fast qPCR Mix, 3 μL of ddH_2_O and 1 μL of forward and reverse primers mixture. Then qRT-PCR was carried out on a CFX connect Real Time PCR Detection System (Bio-Rad, CA, United States). The sequences of all genes and transcription factors in this research were downloaded from EnsemblPlants^1^ and National Center for Biotechnology Information website (NCBI, https://www.ncbi.nlm.nih.gov/. The specific primers (55 genes, 7 transcription factors and actin) were all designed by Primer Premier 5.0, the sequences and information of genes and transcription factors were listed in Supplementary Table 1. The qPCR system was performed using the following program: 95°C for 20 s, then followed by 39 cycles of 95°C for 15 s, finally, 55°C for 15 s and 60°C for 15 s. The ultimate relative expression levels of test genes were calculated by the 2^–△△Ct^ assay.

### Statistical Analysis and Figure Drawing

SPSS 16.0 statistical software package (IBM, Armonk, NY, United States) was used for analyzing results, the data were expressed as the mean ± standard error (SE) of at least three independent replicates. an independent-sample *T*-test was applied to assess significant differences among treatments (*P* < 0.05). Pearson correlation analysis on the variation tendencies between phytohormones and quality-related parameters was also analyzed by SPSS 16.0 (IBM, Armonk, NY, United States) and TBtools (CAN, CHN). GraphPad Prism 9.0 (GraphPad Software Inc., San Diego, CA, United States) and Visio 2020 (Microsoft, SEA, United States) were used for drawing figures.

## Results and Discussion

### The Effect of Exogenous Abscisic Acid on Fruit Quality

**FIGURE 1:**
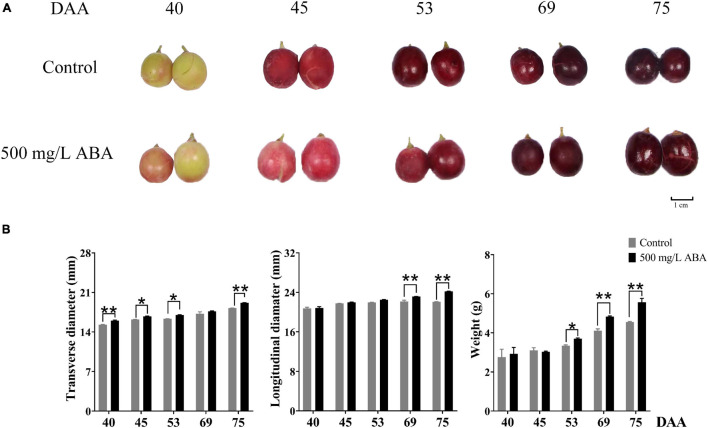
Fruit phenotype and berry appearance parameters. **(A)** Berries collected in two treatment groups (control group and 500 mg/L ABA group) at five sampling stages. DAA 40, November 25th; DAA 45, November 30th; DAA 53, December 8th; DAA 69, December 24th; DAA 75, December 30th. **(B)** Appearance parameters in different sampling times, data from 500 mg/L ABA treatment were represented as a black column while data from control group were represented as a gray column. *Showed the comparatively significant differences with *T*-test (*p* < 0.1, *n* = 3) and **Showed the highly significant differences with *T*-test (*p* < 0.05, *n* = 3).

Physiological parameters in two treatment groups (control, 500 mg/L ABA) measured throughout different collected stages were shown in the Figure 1B. Under 500 mg/L ABA treatment, berry weight in the DAA 75 stage was significantly higher than in the control group (an increasing of 22.4% over the control group), and in the DAA 69 stage the increase rate was 17.5%, berry longitudinal diameters in the DAA 69 and DAA 75 stages were significantly higher than in the control group (an increasing of 4.65 and 9.69% over the control group), while berry transverse diameters in the DAA 40 and DAA 75 stages were significantly higher than in the control group (an increasing of 4.85 and 5.71% over the control group).

**FIGURE 2:**
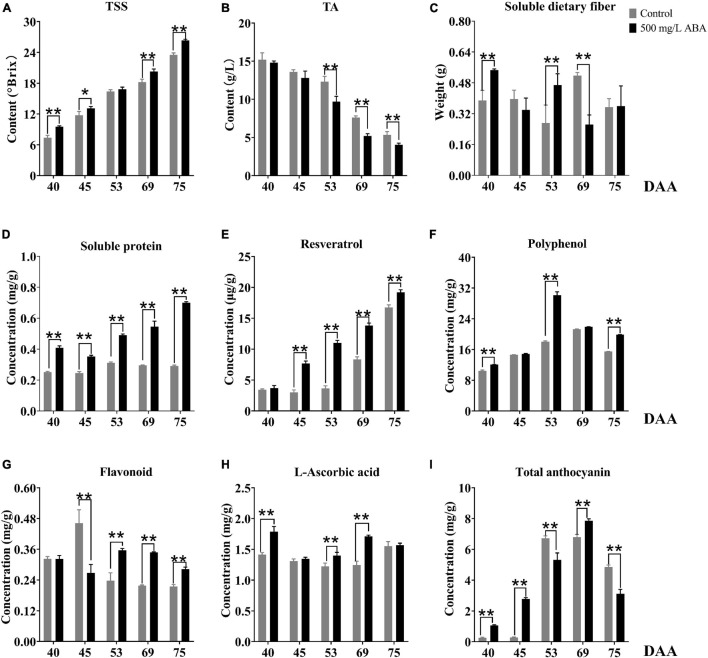
Effect of exogenous ABA treatment on the variation tendency of different quality-related parameters in two treatment groups (control group and 500 mg/L ABA group) at five sampling stages. DAA 40, November 25th; DAA 45, November 30th; DAA 53, December 8th; DAA 69, December 24th; DAA 75, December 30th. **(A)** TSS. **(B)** TA. **(C)** Soluble dietary fiber contents. **(D)** Soluble protein contents. **(E)** Resveratrol contents. **(F)** Polyphenol contents. **(G)** Flavonoid contents. **(H)** L-ascorbic acid contents. **(I)** Total anthocyanin contents. Data from 500 mg/L ABA treatment were shown as a black column while data from control group were shown as a gray column. *Showed the comparatively significant differences with *T*-test (*p* < 0.1, *n* = 3) and **Showed the highly significant differences with *T*-test (*p* < 0.05, *n* = 3).

In this study, we also investigated several interior quality parameters such as TSS, TA, soluble dietary fiber, soluble protein, and resveratrol contents. TSS was shown in the Figure 2A, ABA treatment could significantly increase its contents in the DAA 40, DAA 45, DAA 69, and DAA 75 stages compared to the control group, and the variation tendency was the same in the two groups, it kept rising in all five sampling stages, indicating that the sugar degree continuously increased with fruit ripening. Previous studies suggested that exogenous ABA could significantly increase TSS in “*Crimson Seedless*” grapevine, which was consistent with our study (Ferrara et al., 2013). Meanwhile, we found the differential of TSS levels between the control group and ABA treatment were more and more significant, indicating that exogenous ABA accelerated sugar accumulation in grape fruits mainly occurred in the late ripening period. TA contents decreased gradually with fruit ripening, in the ABA treatment group, its contents were significantly lower than in the control group during the last three sampling stages (Figure 2B). As for soluble dietary fiber contents, we discovered that ABA treatment significantly increased its contents in DAA 40 and DAA 53 stages while significantly decreasing its content in the DAA 69 stage compared with the control group. Interestingly, the variation tendencies in the two groups were the same, it went down first, then it rose and finally it reduced again (Figure 2C). Furthermore, Figure 2D demonstrated that in all five sampling stages, soluble protein contents were confirmed to significantly develop under ABA treatment compared with the control group. Resveratrol, one of the polyphenols, occupy a large amount in grape wine. As a preventive agent for tumor formation, it plays a vital role in the treatment of human cardiovascular and cerebrovascular diseases (Hasan and Bae, 2017). In this study, ABA treatment could significantly increase its concentrations in DAA 45, DAA 53, DAA 69, and DAA 75 stages compared with the control group, and the variation tendency kept increasing in all five sampling stages (Figure 2E).

MDA and Pro are indicators reflecting the degree of stress and ripening on plants (Huan et al., 2016), as shown in the Supplementary Figures 2A,B, MDA contents and Pro contents rose then dropped slightly. Their contents in ABA treatment group were significantly higher than in the control group at DAA 40, 45, 69, 75 stages, indicating that abiotic stress degree and osmotic regulation increased with fruit ripening. The accumulation of a large amount of MDA and Pro would inevitably lead to changes in the contents of CAT, SOD, and POD in plants (Huan et al., 2016). As we expected, Supplementary Figures 2C–E had revealed the consistent variation trends, the activities of the three enzymes all peaked at DAA 53 or 69 stages and then decreased, and ABA treatment could activate their activity at DAA 40, 45, 53, and 75 stages. Interestingly, we found that exogenous ABA could accelerate the emergence of maximum POD value while slowing down the maximum CAT value. It is well-known that reactive oxygen species (ROS) is a general term for a class of chemically active molecules or ions with high oxidative activity, is also an important parameter reflecting the degree of stress faced by fruits (Decros et al., 2019). During fruit development and ripening, ROS content gradually increases and metabolites contributed to stress resistance are accumulated, stress resistance is also correspondingly improved (Giribaldi et al., 2010). ROS produced in plant cells could be eliminated by highly efficient antioxidation-related enzymes (CAT, SOD, POD, APX), and in this process, several antioxidants (phenols, terpenes, thiol derivatives, vitamins, GSH) are enriched accordingly (Decros et al., 2019). Based our obtained results, we speculated that exogenous ABA could effectively eliminate ROS and MDA by regulating the activities of different antioxidant enzymes at different grapevine ripening stages. Another system for scavenging ROS in plants is ascorbate-glutathione cycle, in which APX plays the most important role (Decros et al., 2019). It had been reported that APX could improve the oxidative tolerance of plants and that the activity of GSH (a regulator that maintains cell homeostasis) was positively correlated with APX activity (Wei et al., 2017). In this study, we found that GSH content in both two treatment groups kept rising and under ABA treatment its contents were significant higher in all sampling stages (Supplementary Figure 2F). Moreover, exogenous ABA treatment could improve the activity of APX at all sampling stages and delayed the occurrence of APX peak value, its activity in ABA treatment group reached a maximum at the DAA 53 stage and then slowly declined (Supplementary Figure 2G). The above results demonstrated that GSH and APX increased synergistically at the early stage of fruit ripening and jointly enhanced the antioxidant metabolism process. In addition, DPPH is a very stable nitrogen-centered radical, its scavenging rate could also reflect abiotic stress degree and antioxidant capacity (Decros et al., 2019). Our study further found that the DPPH free radical scavenging rate was gradually increased in both treatment groups, and exogenous ABA treatment significantly increased its ratio at all five sampling stages (Supplementary Figure 2H). The changes of the above enzyme activities under ABA treatment were consistent with research in tomato (Tao et al., 2020), so we made a reasonable inference that during the ripening process of grapevine, plentiful oxidative metabolic reactions occurred and led to ROS, MDA, and DPPH accumulation, the damage of them to cells was reduced by Pro and several antioxidant enzymes, and exogenous ABA treatment contributed to the occurrence of these reactions.

As for antioxidants related to stress resistance, it had been reported that cold plasma treatment led to the accumulation of phenols in pitayas and regulated the ROS signal through the activation of phenylpropane metabolism (Li X. et al., 2019), and exogenous ABA significantly affected grape phenolic composition and contributed to the improvement of stress resistance (Deis et al., 2011). As the results show in Figure 2F, ABA treatment could significantly increase total polyphenol levels in DAA 40, DAA 53, and DAA 75 stages compared with the control group, the variation tendencies both rose first and then dropped down. Therefore, we deduced that exogenous ABA activated ROS signal and regulated the accumulation of polyphenol as the grape fruits ripened. Another vital antioxidant substance in grape is flavonoid, previous study highlighted that exogenous ABA treatment could significantly increase flavonoid content as well as expression levels of key genes revolved in its biosynthesis (Crizel et al., 2020). In our study, we found that ABA treatment could significantly enhance flavonoid concentrations in DAA 53, DAA 69, and DAA 75 stages compared with the control group (Figure 2G). In addition, it had been reported that ABA treatment significantly expedited the metabolism of L-ascorbic acid and improved strawberry antioxidant activity (Li et al., 2015), we found L-ascorbic acid concentrations under ABA treatment were significantly higher in DAA 40, DAA 53, and DAA 69 stages compared with the control group (Figure 2H). The variation tendencies in two groups first decreased and then rose, which was accordant with previous study revealing that L-ascorbic acid accumulated in the late grape ripening stage (Leng et al., 2017). Since UVC induced L-ascorbic acid and phenolic biosynthesis in cherry fruits via increasing mitochondrial activity and ROS (Rabelo et al., 2020), we deduced that exogenous ABA could amplify ROS signal and contributed to the accumulation of several antioxidants at the late grapevine ripening stage to reduce ROS damage to cells, thus delayed the decline of fruit quality due to postmaturity.

Figure 1A had well demonstrated that with fruit development and ripening, the color of grape pericarp constantly deepened, indicating the formation and accumulation of anthocyanins. ABA treatment could significantly increase total anthocyanins contents compared with the control group in the DAA 40, DAA 45, and DAA 69 **stages**. The variation tendency under ABA treatment was consistent with the control group, revealing that it first rose and then dropped down slightly (Figure 2I). Interestingly, we found that in DAA 53 stage anthocyanin content was significantly lower in the treatment group than in the control group (supposedly higher), and exogenous ABA treatment could delay the appearance of anthocyanin peak value. It had been reported that exogenous ABA could promote the postharvest color development of wine grape, and delayed the appearance of the maximum anthocyanin level (Berli et al., 2011). Thus, we deduced although anthocyanin content in the control group might be higher than the treatment group sometimes, the extremely significant effect of ABA on anthocyanins accumulation in late maturity stage was not affected. It had been reported that the long-term effect of ABA spraying on grape berry had been reported not only to influence anthocyanins content but also up-regulate expression levels of key genes in their biosynthesis pathway (Villalobos-González et al., 2016). Results in our study, shown in the Supplementary Figure 1, revealed that in DAA 69 stage under ABA treatment, the expression levels of *VvPAL* (*VIT_11s0016g01520*), *Vv4CL* (*VIT_16s0039g02040*), *VvCHI* (*VIT_13s0067g03820*), and *VvCHS* (*VIT_14s0068g00930*), which were upstream genes in anthocyanins biosynthesis pathway, were significantly up-regulated. Meanwhile, in DAA 40, 53, and 69 stages, the downstream genes in anthocyanins biosynthesis pathway of *VvF3H* (*VIT_04s0023g03370*), *VvF3’5’H* (*VIT_06s0009g02970*), *VvLAR* (*VIT_01s0011g02960*), *VvANR* (*VIT_00s0361g00040*), *Vv3GT* (*VIT_14s0006g03000*), and *Vv5GT* (*VIT_15s0021g00910*) were also up-regulated in the ABA treatment group compared with the control group, especially in the DAA 40 stage, the effect of exogenous ABA was significant. In addition, the expression of transcription factors of VvMYB5a (*VIT_08s0007g07230*), VvMYB14 (*VIT_07s0005g03340*), VvMYCa (*VIT_19s0014g05400*) regulating structural genes for anthocyanins biosynthesis increased under ABA treatment compared to the control group in the last four stages. Interestingly, most of downstream genes in anthocyanins biosynthesis pathway were up-regulated in the DAA 53 stage, while the expression of key genes *VvFLS* (*VIT_18s0001g03430*) related to flavonoids biosynthesis was also increased. Therefore, based on the results that exogenous ABA treatment decreased the total anthocyanin content while increasing flavonoid content in DAA 53 stage, we speculated that exogenous ABA may have accelerated the production of flavonoids or other metabolites during this period. Previous studies had demonstrated that VvMYBA protein interacted with the promoter regions of *UFGT*, *LDOX*, and *CHS3* to mediate the biosynthesis of anthocyanins in grape pericarp (Poudel et al., 2021), PacMYBA interacted with several bHLH transcription factors to activate promoters of *PacDFR*, *PacANS*, and *PacUFGT* (Shen et al., 2014), MdWRKY40 interacted with MdMYB1 to promote anthocyanin biosynthesis, these two proteins were reported tightly in response to ABA signal (An et al., 2019). According to the above findings and our results, we deduced that exogenous ABA could promote the coloration of grape fruits in the late ripening stage, and transcription factors of MYB family could respond to exogenous ABA signals.

### The Effect of Exogenous Abscisic Acid on Endogenous Abscisic Acid Biosynthesis, Catabolism, and Signal Transduction

**FIGURE 3:**
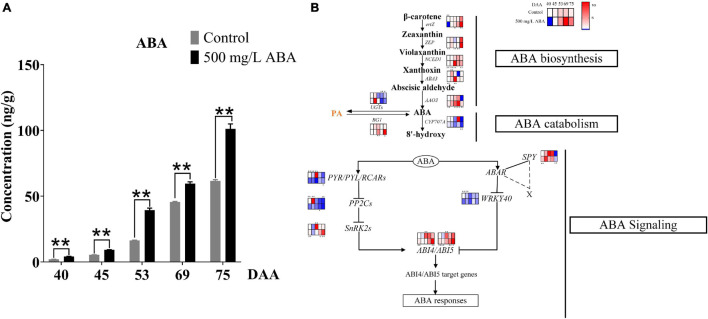
Effect of exogenous ABA treatment on the variation tendency of endogenous ABA contents as well as expression levels of ABA biosynthetic, catabolic, and signaling genes in two treatment groups (control group and 500 mg/L ABA group) at five sampling stages. DAA 40, November 25th; DAA 45, November 30th; DAA 53, December 8th; DAA 69, December 24th; DAA 75, December 30th. **(A)** ABA contents. Data from 500 mg/L ABA treatment were shown as a black column while data from the control group were shown as a gray column. **(B)** Expression of genes and transcription factors involved in ABA biosynthesis, catabolic, and signal transduction Pathways. The relative expression changes in two treatment groups at other stages were relative to the control group (DAA 40) were represented as log2 fold change, key genes (*NCED1*, *ZEP*, *ertZ*, *CYP707A*, *ABA3*, *BG1*, *UGTs*, *AAO3*, *PP2C1*, *SnRK2*, *SPY*, *PYR*) and key transcription factors (WRKY40, ABI4, ABI5) expression levels were shown in the heatmap. heatmap. *Showed the comparatively significant differences with *T*-test (*p* < 0.1, *n* = 3) and **Showed the highly significant differences with *T*-test (*p* < 0.05, *n* = 3).

To explore the effects of exogenous ABA treatment on endogenous ABA biosynthesis, catabolism, and signal transduction, endogenous ABA content was investigated and genes and transcription factors involved in ABA biosynthesis, catabolism, and signal transduction were quantified in all five sampling stages. Results in Figure 3A well revealed that ABA treatment could significantly increase ABA contents compared with the control group in all five sampling stages. The variation tendency of ABA content was going up all the time in both two treatment groups, substantiating that exogenous ABA treatment had a positive feedback effect on the biosynthesis of endogenous ABA in grape berry. ABA biosynthesis was regulated by *NCED1* and *BG1*, Figure 3B well demonstrated that the expression levels of *Vvertz1* (*VIT_02s0025g00240*), *VvNCED1* (*VIT_19s0093g00550*), *VvAAO3* (*VIT_11s0016g03490*), *VvABA3* (*VIT_17s0000g02290*), and *VvBG1* (*VIT_01s0011g00760*) under ABA treatment were significantly higher than in the control group at the DAA 53 stage, these genes were related to the biosynthesis of ABA, while *VvCYP707A* (*VIT_02s0087g00710*) and *VvUGTs* (*VIT_05s0094g01010*) were expressed at a low level in ABA treatment at DAA 40 and 69 stages, these genes contributed to ABA catabolic. Interestingly, the expression levels of *VvZEP* (*VIT_07s0031g00620*) were up-regulated in the ABA treatment group compared to the control group in all five sampling stages. ABA signal transduction also played a crucial role in ABA accumulation and degradation, results in the present study clearly revealed that at the DAA 40 and DAA 75 stages in the ABA treatment group, positive transcription factors of VvABI4 (*VIT_13s0067g01400*) and VvABI5 (*VIT_00s0357g00120*) were significantly up-regulated compared with the control group, while negative transcription factors of VvWRKY40 (*VIT_07s0005g01710*) were down-regulated at four sampling stages except for the DAA 69 stage. Otherwise, in both two treatment groups, the expression of *VvSnRK2* (*VIT_05s0020g01580*) promoting ABA signal transduction were significantly increased at the last two sampling stages, while *VvPP2C* (*VIT_13s0067g01270*) inhibiting ABA signal transduction was significantly decreased at the DAA 40, 45, and 75 stages. Above results revealed that during the late stages of grape ripening in the ABA treatment group, most of the key genes in the ABA biosynthesis pathway were much up-regulated, ABA catabolic genes were down-regulated, and ABA signal transduction was enhanced.

### The Effect of Exogenous Abscisic Acid on Diverse Endogenous Hormones Contents

**FIGURE 4:**
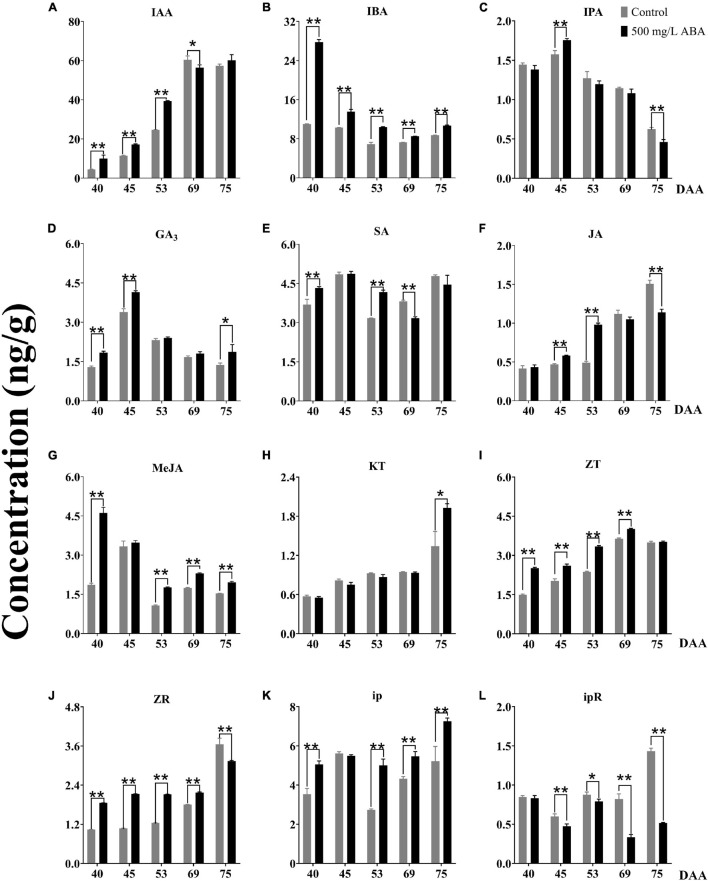
Effect of exogenous ABA treatment on the variation tendency of different phytohormones contents in two treatment groups (control group and 500 mg/L ABA group) at five sampling stages. DAA 40, November 25th; DAA 45, November 30th; DAA 53, December 8th; DAA 69, December 24th; DAA 75, December 30th. **(A)** IAA concentrations. **(B)** IBA concentrations. **(C)** IPA concentrations. **(D)** GA_3_ concentrations. **(E)** SA concentrations. **(F)** JA concentrations. **(G)** MeJA concentrations. **(H)** KT concentrations. **(I)** ZT concentrations. **(J)** ZR concentrations. **(K)** ip concentrations. **(L)** ipR concentrations. Data from 500 mg/L ABA treatment were shown as a black column while data from control group were shown as gray column. *Showed the comparatively significant differences with *T*-test (*p* < 0.1, *n* = 3) and **Showed the highly significant differences with *T*-test (*p* < 0.05, *n* = 3).

It has been currently recognized that phytohormones play an important regulatory role in almost all plant growth and development processes, especially in regulating fleshy fruits quality improvement (Kumar et al., 2013). To obtain further investigation of variation trends of other phytohormones under exogenous ABA treatment, 12 kinds of phytohormones including IAA, SA, GA_3_, JA, MeJA, IBA, IPA, ZT, ZR, ip, ipR, and KT contents in both two treatment groups were detected. The results sufficiently demonstrated that under ABA treatment, IAA concentrations were significantly enhanced in the DAA 40, DAA 45, and DAA 53 stages compared with the control group, the variation tendency in the ABA treatment group was going up all the time while the variation tendency in the control group first went up then decreased (Figure 4A). Exogenous ABA treatment indeed reduced the content of IAA as the development of peach fruit (Liu, 2019), while improving kiwi’s ability to resist drought stress via increasing IAA content (Wang et al., 2011). Thus, a conjecture could be put forward that the biosynthesis of endogenous IAA could respond to exogenous ABA signal, exogenous ABA treatment had positive feedback effect on the endogenous IAA biosynthesis at early grape ripening stages, while this effect was not obvious at the later stage of fruit ripening. IBA and IPA are precursors in the IAA synthetic pathway, we found that IBA concentrations under ABA treatment were significantly higher than in the control group in all five sampling stages (Figure 4B), while IPA concentration under ABA treatment was only significantly higher than in the control group in the DAA 45 stage (Figure 4C). However, the variation tendencies of two hormones were opposite to the ABA variation tendency, two of them decreased gradually as the fruit ripened. Due to the fact that ABA, IBA, and IPA were antagonistic to each other and jointly regulated flowering (Pal, 2019), we inferred that ABA antagonism of IBA and IPA regulated fruit quality improvement. Furthermore, we found that ABA treatment could significantly increase GA_3_ contents compared with the control group in the DAA 40, DAA 45, and DAA 75 stages (Figure 4D), which was consistent with previous studies indicating that ABA treatment could significantly increase GA contents in early sweet cherry development (Gutiérrez et al., 2021). SA concentration did not change significantly throughout the maturity and it was higher in grapevine at the early ripening stage (Figure 4E), the variation tendencies of GA_3_ and SA were the same, first went up, then went down, and finally went up. The above results were related to previous studies, revealing endogenous ABA and SA co-regulated ABA signal transduction (Wang et al., 2015). Otherwise, both JA and MeJA concentrations under ABA treatment were higher than in the control group, but the change trends were showed distinctly opposite (Figures 4F,G), which was consistent with previous study confirming JA and MeJA were antagonistic in watermelon ripening (Kojima et al., 2021). The variation trends of JA and ABA in strawberry during ripening had been reported (Lee et al., 2020), while the change of MeJA under ABA treatment was not clear. Based on our results, we deduced that JA and MeJA came into play at different developmental stages of grapevine, and exogenous ABA positively regulated JA while negatively regulating MeJA.

Cytokinins (CTKs) generally synthesize in plant roots, have positive effects on promoting differentiation and growth of plant tissues, and play notable roles in regulating the development and sex differentiation of plants (Mok, 2019). In our study, KT concentration was higher under ABA treatment than in the control group at the DAA 75 stage, the variation trends were increasing all the time (Figure 4H). Under ABA treatment, ZT and ZR concentrations were significantly higher than the control group in the first four sampling stages (Figures 4I,J), while iP contents were significantly higher and ipR contents were conspicuously lower than in the control group in the last two sampling stages (Figures 4K,L). It had been reported that exogenous ABA could up-regulate the contents of ZT and ZR in the early ripening stage of sweet cherry (Gutiérrez et al., 2021), while other CTKs were not discussed. Therefore, we deduced that ZT and ipR regulated by exogenous ABA would accumulate at the veraison stage while ip, ZR, and KT regulated by exogenous ABA might accumulate at the maturity stage.

Exogenous IAA and ABA co-regulated the improvement of strawberry berry enlargement (Chen et al., 2016), these two phytohormones were closely related to the improvement of antioxidant activity in blueberry, while the improvement of fruit flavor was influenced by the increase of ABA and SA contents (Aires et al., 2017). In our study, we discovered that under exogenous ABA treatment and with grapevine ripening, berry enlargement was accompanied by the increase of endogenous IAA and KT contents, and the accumulation of ABA, SA, and ip was accordant with the enrichment of antioxidant substances at the late stage of fruit ripening. Thus, we legitimately inferred that exogenous ABA could affect the biosynthesis of endogenous phytohormones, fruit exterior quality improvement was regulated by endogenous IAA and KT, and stress resistance improvement was mediated by endogenous ABA, SA, and CTKs during grapevine developmental and ripening stages.

### Exogenous Abscisic Acid Regulated Sugars Accumulation via Mediating Endogenous Hormones Biosynthesis

**FIGURE 5:**
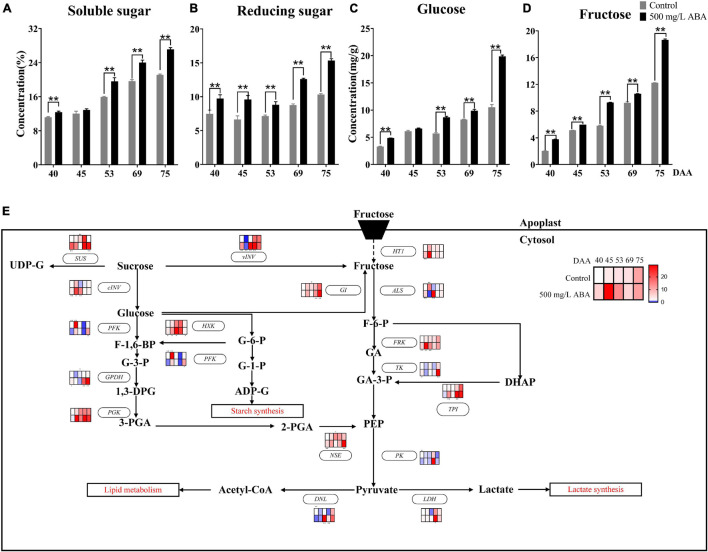
Effect of exogenous ABA treatment on the variation tendency of different sugars contents as well as expression levels of sugars biosynthetic and catabolic genes in two treatment groups (control group and 500 mg/L ABA group) at five sampling stages. DAA 40, November 25th; DAA 45, November 30th; DAA 53, December 8th; DAA 69, December 24th; DAA 75, December 30th. **(A)** Soluble sugar levels. **(B)** Reducing sugar levels. **(C)** glucose levels. **(D)** fructose levels. Data from 500 mg/L ABA treatment were shown as black column while data from control group were shown as gray column. **(E)** Expression of genes involved in sugars biosynthesis and catabolic pathways. The relative expression changes in two treatment groups at other stages were relative to the control group (DAA 40) were represented as log2 fold change. The relative expression levels of key genes (*HT1*, *ALS*, *FRK*, *TK, PK*, *GI*, *vVIN*, *SUS*, *cINV, PGK, TPI, GPDH, NSE*, *DNL*, *LDH, PFK*, *HXK*) were shown as heatmap. *Showed the comparatively significant differences with *T*-test (*p* < 0.1, *n* = 3) and **Showed the highly significant differences with *T*-test (*p* < 0.05, *n* = 3).

There were three main types of sugars in grapevine, including fructose, glucose, and sucrose (Liu et al., 2007). Due to the low concentration sucrose has in the grape berry, only fructose and glucose in grape juice were quantified by HPLC in this research. As results show in Figures 5A–D, soluble sugar, reducing sugar, glucose, and fructose levels displayed the same variation tendency, they all kept rising in all 5 sampling stages, and ABA treatment significantly increased their levels in the DAA 40, DAA 53, DAA 69, and DAA 75 stages, which was accordant with previous study indicating that the maturity stage was a key period of sugar enrichment (Shen et al., 2017).

Crosstalk in the sugar and ABA signaling pathway had also been well studied, affirming that *VvSK1* and *VvHT1* could responded to ABA signal and regulated hexose transport and transmission (Lecourieux et al., 2010), grape hexokinase was involved in ABA regulation of sucrose synthase expression, indicating there existed crosstalk between ABA, hexose, and sucrose (Wang et al., 2017). Previous study also reported that FaSigE (a positive regulator of glucose catabolism) interacted with FaABAR (ABA signaling receptor) and actively regulated strawberry ripening (Zhang et al., 2017). In this study, the effects of exogenous ABA on glucose biosynthesis and catabolism were discussed at the transcriptional level (Figure 5E), revealing that under ABA treatment at DAA 69 and DAA 75 stages, the expression levels of genes involved in fructose catabolism including *VvALS* (*VIT_16s0022g01030*), *VvTK* (*VIT_17s0000g08560*), *VvPK* (*VIT_08s0007g04170*), and *VvFRK* (*VIT_01s0011g00240*) were up-regulated, while genes participating in glucose catabolism including *VvPFK* (*VIT_14s0108g00540*), *VvGPDH* (*VIT_14s0219g00280*), *VvPGK* (*VIT_19s0085g00380*), *VvHXK* (*VIT_18s0001g14230*) were expressed higher than in the control group. Otherwise, compared with the control group, in the DAA 69 and 75 stages, *VvGI* (*VIT_18s0001g07280*) contributing to the conversion of glucose to fructose were expressed at significant higher level, while the expression of *VvHT1* (*VIT_00s0515g00050*) referred to be responsible for transporting hexose from apoplast to cytosol was increased in four sampling stages except for DAA 53 stage. Thus, it could be deduced that fructose was accumulated due to the continuous conversion of glucose and sucrose in the developmental stages of grapevine. Furthermore, we found that the expression of genes contributing to the conversion of sucrose to glucose and fructose including *VvvINV*, *VvcINV* (*VIT_06s0061g01520*) were all up-regulated under ABA treatment in all sampling stages. The above results attested that grape ripening was accompanied by a marked accumulation of sugars, and their biosynthesis and catabolism would respond to exogenous ABA signal.

It has been reported that IAA and SA might mediate sugar accumulation in tomato, which demonstrated there existed interaction between IAA, SA, and sugars (Malik and Lal, 2018). In this study, due to total sugar content in grape berry continuously increasing, SA content was higher in the early ripening period, while IAA and ABA content was higher in the late ripening period, thus we speculated that the sugar accumulation in the early stage of grape ripening was mainly regulated by endogenous SA, while the sugar accumulation in the late stage of grape ripening was mainly regulated by endogenous ABA and IAA, and the changes of these endogenous phytohormones were induced by exogenous ABA.

### Exogenous Abscisic Acid Enriched Fatty Acids in Berry via Regulating Endogenous Hormones Biosynthesis

**FIGURE 6:**
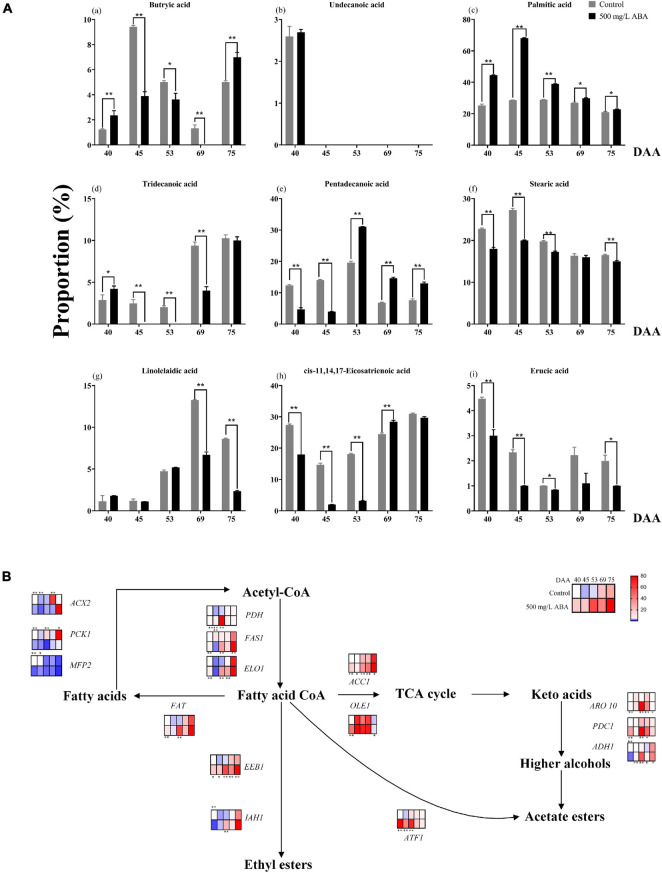
Changes in the proportion of fatty acids as well as expression levels of fatty acids biosynthetic and catabolic genes in two treatment groups (control group and 500 mg/L ABA group) at five sampling stages. DAA 40, November 25th; DAA 45, November 30th; DAA 53, December 8th; DAA 69, December 24th; DAA 75, December 30th. **(A)** The proportions of fatty acids in grapevine at different sampling time. **(a)** Butryic acid. **(b)** Undecanoic acid. **(c)** Palmitic acid. **(d)** Tridecanoic acid. **(e)** Pentadecanoic acid. **(f)** Stearic acid. **(g)** Linolelaidic acid. **(h)** *cis*-11,14,17-Eicosatrienoic acid. **(i)** Erucic acid. **(B)** Expression of genes involved in fatty acids biosynthesis and catabolic pathways. The relative expression changes in two treatment groups at other stages were relative to the control group (DAA 40) were represented as log2 fold change. Key genes (*ACC1, PDH, FAS1, FAT, ELO1, IAH1, OLE1, ADH1, ARO10, PDC1, ACX2, PCK1, MFP2, ATF1, EEB1*) expression levels were shown as heat map. *Showed the comparatively significant differences with *T*-test (*p* < 0.1, *n* = 3) and **Showed the highly significant differences with *T*-test (*p* < 0.05, *n* = 3).

Lipids are important nutrients in plants and animals, which have crosstalk with the biosynthesis of many metabolites and play an important role in plant stress resistance and flavor formation (Liu et al., 2019). Although lipids play an important role in regulating quality improvement, the understanding of the effect of exogenous ABA on these substances in grape fruits and the variation trend of fatty acids ratio during fruit ripening is much lacking. To thoroughly make inquiry into different types of Fas proportion in both ABA treatment group and the control group, a total of nine Fas including butryic acid (C_4_:0), undecanoic acid (C_11_:0), tridecanoic acid (C_13_:0), pentadecanoic acid (C_15_:0), palmitic acid (C_16_:0), stearic acid (C_18_:0), linolelaidic acid (C_18_:2n6t), *cis*-11,14,17-eicosatrienoic acid (C_20_:3n3), and erucic acid (C_22_:1n9) were measured in all five sampling stages. As shown in the Figures 6Aa–i, the variation tendency of butyric acid in two groups was same: first up, then down, and then up again, only at the DAA 40 and 75 stages was exogenous ABA significantly promoted in its production. Undecanoic acid proportion was relatively small, and none of the last four sampling stages were detected, leading to speculation that their contribution to fruit quality improvement was small. Moreover, palmitic acid was the main FAs in grapevine, which occupied the largest proportion in all five sampling stages, exogenous ABA treatment could significantly contribute to the increase of its proportion. And the proportion of tridecanoic acid was higher under ABA treatment than in the control group only in the DAA 40 stage, while in other stages had contrary results. Interestingly, the variation tendencies of pentadecanoic acid, stearic acid, and linolelaidic acid were consistent: first up, and then down, which indicated that they might accumulate in the early fruit ripening. Exogenous ABA could significantly promote pentadecanoic acid proportions in the last three sampling stages, while the stearic acid proportions under ABA treatment were significantly smaller than the control group in four stages except for the DAA 69 stage. Linolelaidic acid proportions accounting for the whole FAs under ABA treatment were larger than the control group in the DAA 40 and 53 stages, and other stage results showed inversely. Otherwise, *cis*-11,14,17-eicosatrienoic acid and erucic acid showed consistent variation tendencies: first down, and then up, indicating that they might accumulate in the late fruit ripening. We found that exogenous ABA treatment significantly inhibited *cis*-11,14,17-eicosatrienoic acid biosynthesis in the first three sampling stages while inhibiting erucic acid biosynthesis in all sampling stages. In summary, undecanoic acid, palmitic acid, stearic acid, and erucic acid occupied a large proportion in the early fruit ripening, while butyric acid, tridecanoic acid, pentadecanoic acid, linolelaidic acid, and *cis*-11,14,17-Eicosatrienoic acid would accumulate in the late fruit ripening, exogenous ABA had great effect on their proportion changes (With the exception of palmitic acid and pentadecanoic acid, most of the other effects are inhibitory). Due to the fact that FAs content in jujube fruits changed constantly during ripening and palmitic acid had the most obvious effect on the fruit flavor improvement (Fu et al., 2021). Thus, we concluded that exogenous ABA promoted the improvement of grape fruit flavor and nutritional value via promoting palmitic acid enrichment and regulating other FAs proportions.

Fas, as a key intermediate in the ABA, anthocyanins, sugars biosynthesis, and catabolism pathways, the change of its content will also affect the biosynthesis of several nutrients (Lafuente et al., 2021). It is necessary to explore the differential genes that exogenous ABA regulates FAs metabolism at the molecular level. As results in Figure 6B exhibited, in the last three sampling stages, the expression levels of *VvFAT* (*VIT_05s0094g00930*), *VvIAH1* (*VIT_10s0003g00960*), *VvFAS1* (*VIT_01s0011g06640*), *VvELO1* (*VIT_04s0023g01140*), *VvOLE1* (*VIT_14s0066g00700*), *VvADH1* (*VIT_18s0001g15410*), *VvARO10* (*VIT_03s0038g02120*), *VvPDC1* (*VIT_13s0067g00340*), *VvATF1* (*VIT_16s0039g00570*), and *VvEEB1* (*VIT_11s0016g01930)* were up-regulated under ABA treatment compared with the control group, these genes were responsible for FAs and other aromatic substances biosynthesis. At the DAA 40 stage, the expression levels of *VvACX2* (*VIT_00s0662g00010*), *VvPCK1* (*VIT_07s0205g00070*), and *VvMFP2* (*VIT_05s0077g02140*) were significantly down-regulated in the ABA treatment group, these genes contributed to the catabolism of FAs. The above results indicated that exogenous ABA could have contributed to FAs accumulation and flavor improvement. Interestingly, under exogenous ABA treatment, the expression of *VvACC1* (*VIT_12s0059g01380*) was significantly increased in all sampling stages compared with the control group, indicating that plenty of fatty acid CoA entered the TCA cycle to participate in the formation of other important aromatic substances, and this process was regulated by exogenous ABA. Thus, we deduced that FAs accumulation would respond to exogenous ABA signal, however, the specific molecular mechanism of how it regulates the biosynthesis of quality-related metabolites in response to ABA signal remains to be further explored.

Plant hormones interaction plays an important role in the ripeness and lipids enrichment in fleshy fruits, GA and ABA interplayed in the regulation of fruit ripening in avocados (Vincent et al., 2020), and MYB41, MYB107, and MYC2 promoted ABA-mediated primary fatty alcohol accumulation in kiwi fruit by activating AchnFAR (Wei et al., 2020). In this study, GA_3_ content was higher in the early stage of fruit ripening while ABA content was higher in the late ripening stage, accompanied by the continuous changes in the proportion of FAs and the accumulation of FAs. Consequently, it could be deduced that exogenous ABA mediated the accumulation of endogenous ABA and GA_3_ at different developmental stages, thereby contributing to the enrichment of FAs.

### Correlation Analysis of Different Endogenous Phytohormones and Quality-Related Parameters

**FIGURE 7:**
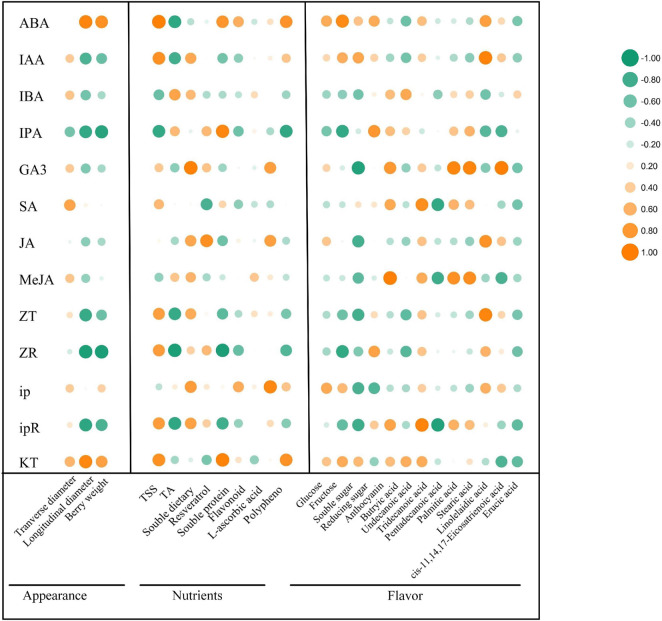
Correlation analysis of different phytohormones with appearance parameters, nutrients and flavor-related metabolites. The vertical row represented 13 phytohormones and the horizontal row represented 3 kinds of appearance parameters, 8 kinds of nutrients and 14 kinds of flavor-related metabolites. The size of the bubbles indicated the strength of the relational degree, with orange representing a positive correlation and green representing a negative correlation.

IAA, GA, and CTK are the main regulators of fruit ripening (Chen et al., 2016). Berry enlargement and fruit ripening depends on IAA, CTKs, and GA, pericarp coloration of climactic and non-climactic fruits is controlled via ETH-dependent or ETH-independent manner (Kumar et al., 2013). Since the correlation between the changes of endogenous phytohormones regulated by exogenous ABA and the improvement of grape fruit quality has not been discussed, it is of great significance to explore the relationship between different endogenous phytohormones variation and berry enlargement, pericarp coloration, sugars accumulation, flavor formation, as well as stress resistance enhancement. To further validate our hypothesis, correlation analysis was carried out on the changes of 13 kinds of endogenous hormones and 25 kinds of quality-metabolites (Figure 7). The results revealed that under exogenous ABA treatment, the changes of ABA and KT were relatively associated with fruit appearance and quality improvement, the change of IAA was tightly correlated with sugars, anthocyanins, linolelaidic acid, and *cis*-11,14,17-eicosatrienoic acid accumulation. Meanwhile, we also found that the change of ip was closely related to berry enlargement, sugars accumulation, and antioxidant activity improvement. Meanwhile, the change of GA_3_ had the greatest effect on the enrichment of FAs, including butyric acid, tridecanoic acid, palmitic acid, stearic acid, and *cis*-11,14,17-eicostrienoic acid. Therefore, we concluded that the endogenous biosynthesis of ABA, IAA, and CTKs regulated by exogenous ABA was most closely related to fruit quality improvement and stress resistance enhancement during grape fruit development and ripening.

## Conclusion

To sum up, this research certified that exogenous ABA treatment had crucial effects on berry size, endogenous hormone contents variation, sugars formation, anthocyanins accumulation, and FAs enrichment. The correlation analysis of diverse phytohormones and quality-related parameters confirmed that ABA/KT was correlated with berry size, ABA/IAA/KT was correlated with sugar accumulation, ABA/IPA/ZR was correlated with anthocyanin accumulation, and GA3/MeJA was correlated with FAs enrichment. Thus, we deduced that endogenous hormones with different physiological functions could respond to exogenous ABA signal, although these phytohormones had different contributions to the improvement of fruit quality, they ultimately regulated the overall improvement of grape fruit quality. Therefore, spraying 500 mg/L ABA during the grapevine veraison stage was indeed an effective means to improve grape fruit quality, which is likely to have a broad prospect in future cultivation. However, further research on exploring the specific molecular mechanism of exogenous ABA regulating fruit quality improvement via inducing endogenous phytohormone biosynthesis should be carried out.

## Data Availability Statement

The original contributions presented in the study are included in the article/Supplementary Material, further inquiries can be directed to the corresponding author/s.

## Author Contributions

JL carried out all the experiments, data analyzations, manuscript writing, and figures drawing. BL provided the experimental methods and ideas, collected, grinded samples as well as reviewed the manuscript. DL collected the samples. JH and YZ provided experimental materials, CM, WX, SJ, CZ, and SW reviewed the manuscript. XL and LW designed the experiments, reviewed the manuscript, and supervised this study. All authors contributed to the article and approved the submitted version.

## Conflict of Interest

The authors declare that the research was conducted in the absence of any commercial or financial relationships that could be construed as a potential conflict of interest.

## Publisher’s Note

All claims expressed in this article are solely those of the authors and do not necessarily represent those of their affiliated organizations, or those of the publisher, the editors and the reviewers. Any product that may be evaluated in this article, or claim that may be made by its manufacturer, is not guaranteed or endorsed by the publisher.

## Footnotes

1
http://plants.ensembl.org/info/about/index.html

## Supplementary Material

Supplementary Figure 1 Effect of exogenous ABA treatment on the expression levels of anthocyanin biosynthetic and catabolic genes in two treatment groups (control group and 500 mg/L ABA group) at 5 sampling stages. DAA 40, November 25th; DAA 45, November 30th; DAA 53, December 8th; DAA 69, December 24th; DAA 75, December 30th. **(A)** Expression of genes involved in anthocyanin biosynthesis pathway. The relative expression levels of key genes (*PAL*, *4CL*, *CHI*, *CHS*, *F3H*, *F3’5’H*, *FLS*, *DFR*, *LDOX*, *3GT*, *5GT*, *ANR*, *LAR*) were represented as heatmap. **(B)** Heatmap of key transcription factors (MYB5a, MYB5b, MYCa, MYB14) in anthocyanin biosynthesis pathway. The relative expression changes in two treatment groups at other stages were relative to the control group (DAA 40) were represented as log2 fold change. ^∗^Showed the comparatively significant differences with *T*-test (*p* < 0.1, *n* = 3) and ^∗∗^Showed the highly significant differences with *T*-test (*p* < 0.05, *n* = 3).

Supplementary Figure 2 Effect of exogenous ABA treatment on stress-related parameters and antioxidant enzyme activity in two treatment groups (control group and 500 mg/L ABA group) at 5 sampling stages. DAA 40, November 25th; DAA 45, November 30th; DAA 53, December 8th; DAA 69, December 24th; DAA 75, December 30th. **(A)** MDA concentrations. **(B)** Pro concentrations. **(C)** POD activity. **(D)** CAT activity. **(E)** SOD activity. **(F)** GSH concentrations. **(G)** APX activity. **(H)** DPPH free radical scavenging rate. Data from 500 mg/L ABA treatment were shown as black column while data from control group were shown as gray column. ^∗^Showed the comparatively significant differences with *T*-test (*p* < 0.1, *n* = 3) and ^∗∗^Showed the highly significant differences with *T*-test (*p* < 0.05, *n* = 3).

Supplementary Table 1 Primers for qRT-PCR quantification. All primers were designed by Primer Premier 5.0.

Supplementary Table 2 Detection and quantification limits of target compounds by HPLC.
