# Supplementary figures and images for: Exogenous Abscisic Acid Mediates Berry Quality Improvement by Altered Endogenous Plant Hormones Level in “Ruiduhongyu” Grapevine
Source: Front Plant Sci. 2021 Oct 1;12:739964. doi: 10.3389/fpls.2021.739964 (PMC8519001; doi:10.3389/fpls.2021.739964)

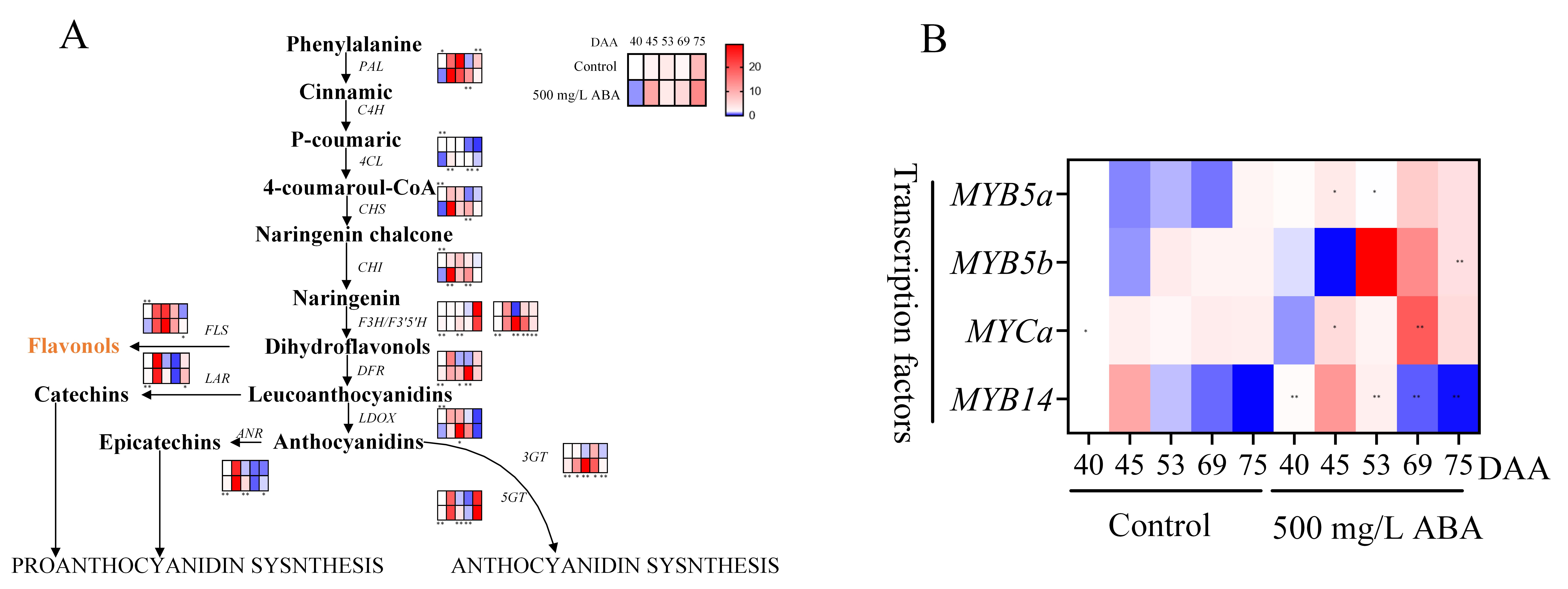

Supplement: Supplementary Figure 1 — Effect of exogenous ABA treatment on the expression levels of anthocyanin biosynthetic and catabolic genes in two treatment groups (control group and 500 mg/L ABA group) at 5 sampling stages. DAA 40, November 25th; DAA 45, November 30th; DAA 53, December 8th; DAA 69, December 24th; DAA 75, December 30th. (A) Expression of genes involved in anthocyanin biosynthesis pathway. The relative expression levels of key genes (PAL, 4CL, CHI, CHS, F3H, F3’5’H, FLS, DFR, LDOX, 3GT, 5GT, ANR, LAR) were represented as heatmap. (B) Heatmap of key transcription factors (MYB5a, MYB5b, MYCa, MYB14) in anthocyanin biosynthesis pathway. The relative expression changes in two treatment groups at other stages were relative to the control group (DAA 40) were represented as log2 fold change. ∗Showed the comparatively significant differences with T-test (p < 0.1, n = 3) and ∗∗Showed the highly significant differences with T-test (p < 0.05, n = 3). [file Image_1.jpg]

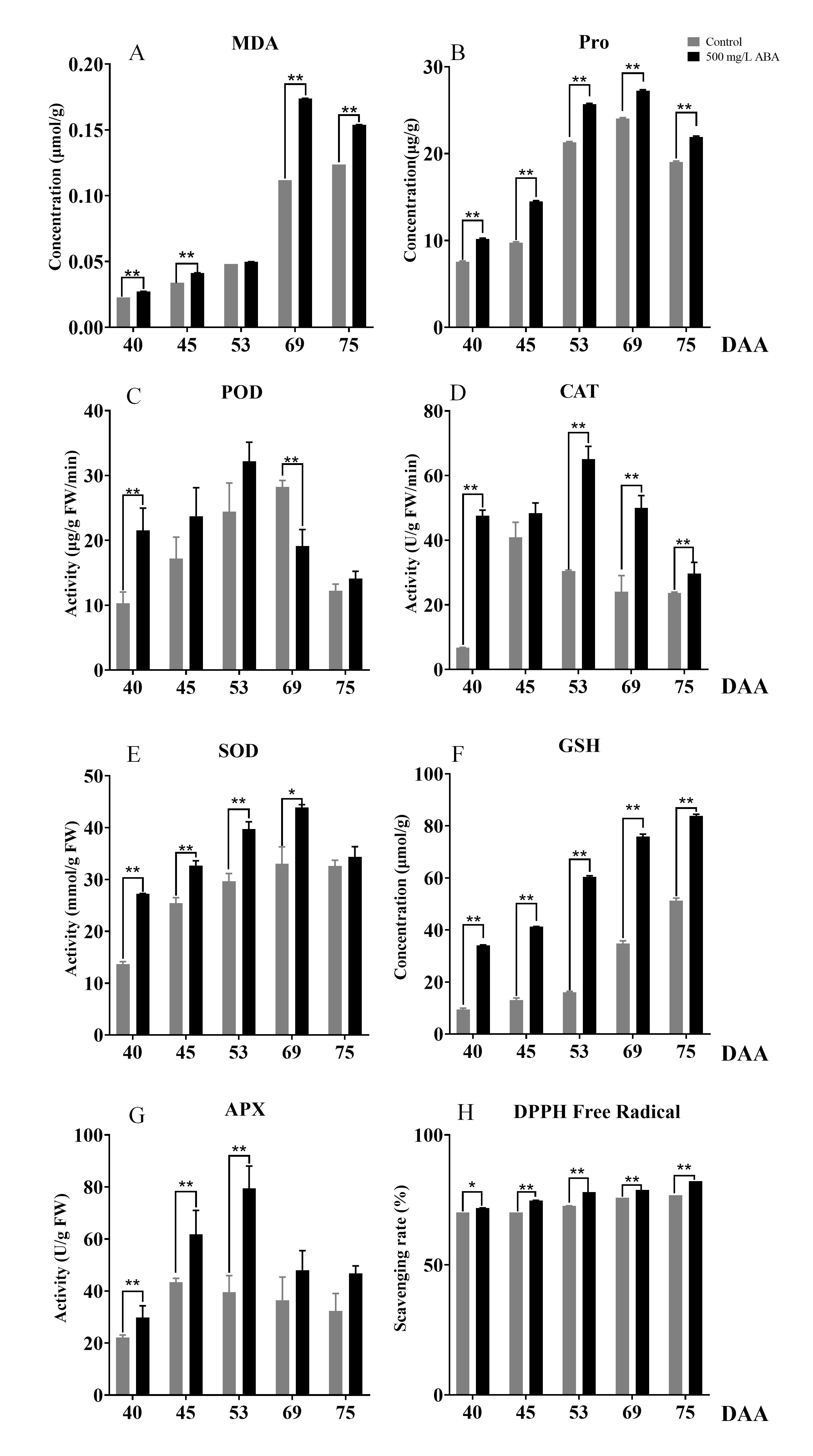

Supplement: Supplementary Figure 2 — Effect of exogenous ABA treatment on stress-related parameters and antioxidant enzyme activity in two treatment groups (control group and 500 mg/L ABA group) at 5 sampling stages. DAA 40, November 25th; DAA 45, November 30th; DAA 53, December 8th; DAA 69, December 24th; DAA 75, December 30th. (A) MDA concentrations. (B) Pro concentrations. (C) POD activity. (D) CAT activity. (E) SOD activity. (F) GSH concentrations. (G) APX activity. (H) DPPH free radical scavenging rate. Data from 500 mg/L ABA treatment were shown as black column while data from control group were shown as gray column. ∗Showed the comparatively significant differences with T-test (p < 0.1, n = 3) and ∗∗Showed the highly significant differences with T-test (p < 0.05, n = 3). [file Image_2.jpg]

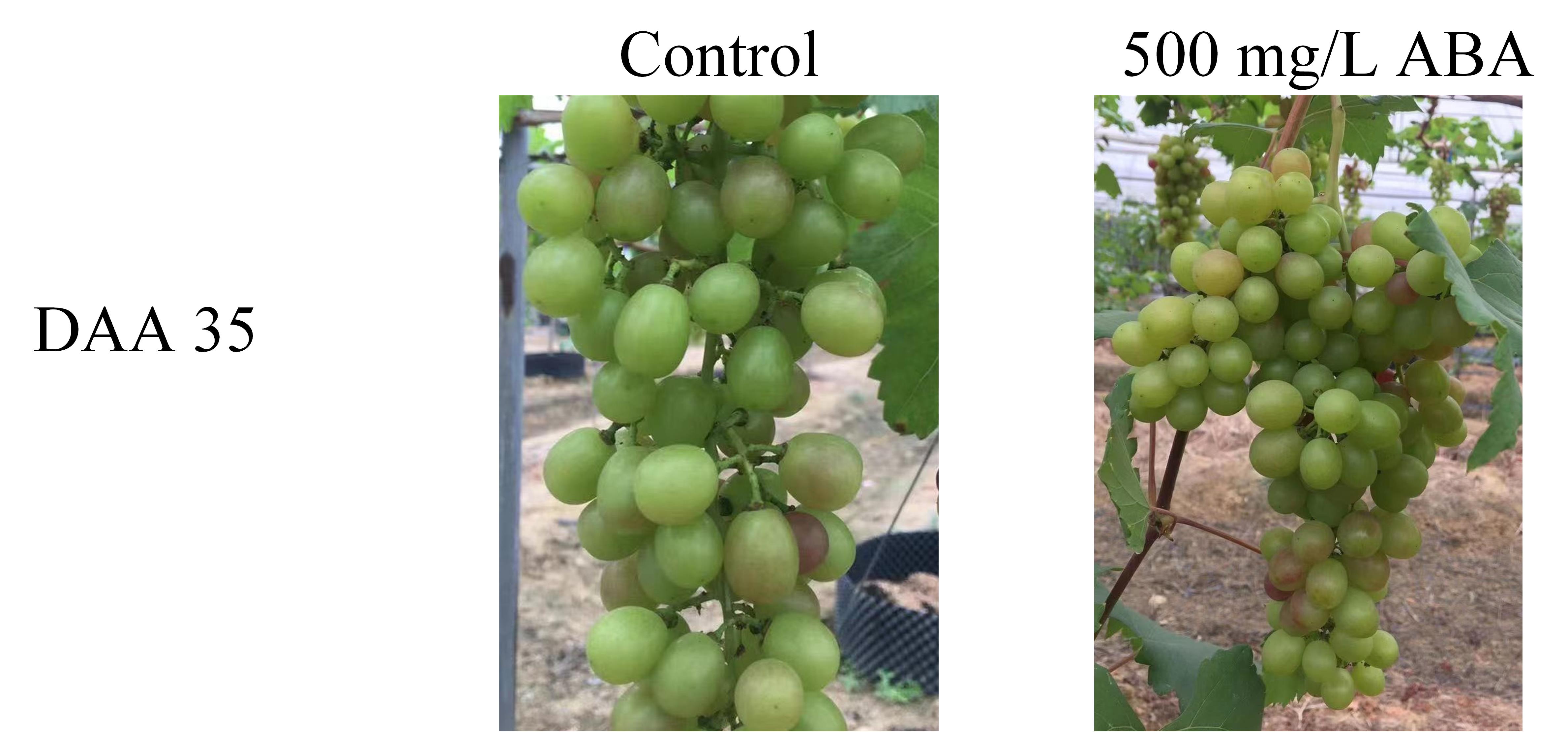

Supplement: Supplementary file 5 [file Image_3.jpg]
