# Supplementary material for: Exogenous Abscisic Acid Mediates Berry Quality Improvement by Altered Endogenous Plant Hormones Level in “Ruiduhongyu” Grapevine
Source: Front Plant Sci. 2021 Oct 1;12:739964. doi: 10.3389/fpls.2021.739964 (PMC8519001; doi:10.3389/fpls.2021.739964)
Supplement: Supplementary Table 1 — Primers for qRT-PCR quantification. All primers were designed by Primer Premier 5.0. [file Table_1.docx]

| **Target genes** | **Forward sequence（5’ → 3’）** | **Reverse Sequence（5’ → 3’）** |
| --- | --- | --- |
| *VvPAL (VIT_11s0016g01520)* | CTCACACCACAACGGCAACG | CGCCACCATTCTCTTCACCTC |
| *Vv4CL (VIT_16s0039g02040)* | ACCACCTCCCTCTCCACAC | ACCACCTCCCTCTCCACAC |
| *VvCHS (VIT_14s0068g00930)* | AGCCAGTGAAGCAGGTAGCC | GTGATCCGGAAGTAGTAAT |
| *VvCHI (VIT_13s0067g03820)* | AGACTGTGGAGGAGTTAGCG | AGAATGGAGTTGCCTGGTG |
| *VvF3H (VIT_04s0023g03370)* | CCAATCATAGCAGACTGTCC | TCAGAGGATACACGGTTGCC |
| *VvF3'5'H (VIT_06s0009g02970)* | ACTAAGCCACAGGAAACTAA | AAACCGCTCAGACCAAAACC |
| *VvFLS (VIT_18s0001g03430)* | AAACCACCTACTTACAGAGC | ACCTAACCCCAGTGACAGAC |
| *VvDFR (VIT_18s0001g12800)* | GAAACCTGTAGATGGCAGGA | GGCCAAATCAAACTACCAGA |
| *VvLDOX (VIT_02s0025g04720)* | ACTCTTTGGGGATTGACTGG | AGGGAAGGGAAAACAAGTAG |
| *VvLAR (VIT_01s0011g02960)* | TGCTTTTGTGATTTTGTTAGAGG | CCCTTCCCCGATTGAGAGTA |
| *Vv3GT (VIT_14s0006g03000)* | GGGATGGTAATGGCTGTGG | ACATGGGTGGAGAGTGAGTT |
| *Vv5GT (VIT_15s0021g00910)* | TTCCATGGCTGAACTCAAAAC | AACATCCAACTGCTTGGTGAC |
| *VvANR (VIT_00s0361g00040)* | CAATACCAGTGTTCCTGAGC | AAACTGAACCCCTCTTTCAC |
| *VvMYB5a (VIT_08s0007g07230)* | CATGTCTCCCTGAAAATGATGA | TGCAAGGATCCATTTCACATAC |
| *VvMYB5b (VIT_04s0008g03790)* | GGTGTTCTTTAATTTGGCTTCA | CACAACAACACAACCACATACA |
| *VvMYCa (VIT_19s0014g05400)* | TGCTGTGGCTGTGAGGAGTATC | CTCTTGGACTGTTGGTCGGTTT |
| *VvMYB14 (VIT_07s0005g03340)* | GGTCCTTGATTGCGGGTAG | TGTCATCATTCGGTTGTGG |
| *Vvactin* | GACAATTTCCCGTTCAGCAGT | GATTCTGGTGATGGTGTGAGT |
| *VvNCED1 (VIT_19s0093g00550)* | CGTGGCTCTCCTGTGGTGT | GCCTTCGTCGCATTCGTTG |
| *VvPP2C1 (VIT_13s0067g01270)* | CCACGCTTCCCTCAAACCCTCCCA | CATCCACCACTCCATCATCCCCGC |
| *VvABA3 (VIT_17s0000g02290)* | CGAGATGAATGGAGCAGGGAG | TGGACTTGGAGTCGAAGCAGA |
| *VvCYP707A (VIT_02s0087g00710)* | GTCTGAACCAACTGTCAACGA | TGAAAGGAGTGTGAGAACGCA |
| *VvAAO3 (VIT_11s0016g03490)* | AAGTGAGGTGGAGGTAAAT | ACTGCTTGGGTATGGTGTC |
| *Vvertz (VIT_02s0025g00240)* | GCTATCGTTGCTGTCTACTACA | CACAGAAAGAGCAAATGTACCC |
| *VvZEP (VIT_07s0031g00620)* | GATACAGAGTAATGCATTGGCG | CGCAGGAGTGAATGTATCAAAC |
| *VvBG1 (VIT_01s0011g00760)* | GCAATCAAGGATGGAGTTGATG | GAAACGTTTGGTGTAACCTTGA |
| *VvUGTs (VIT_05s0094g01010)* | GGATTGGTATAGGAGTTGAGGG | TCTAGCAAGCTCTTTTAGGACC |
| *VvSVP (VIT_03s0167g00070)* | GATTTAGCTCCATTCGTGCAAT | TTTCCCAATAGAGCACCTTGAT |
| *VvSnRK2 (VIT_05s0020g01580)* | CAATGCATGCTTTGAAAGTGTG | GCCAAACTGCAGTCTGATTTTA |
| *VvABI4 (VIT_13s0067g01400)* | GAACAAGACTGTGGATGAGTTG | CCGTTCATTATCACATCCACAC |
| *VvABI5 (VIT_00s0357g00120)* | CAGAATGGTAGTACTGCAAACG | ACCCTCGAAAACTGATTCAGAA |
| *VvWRKY40 (VIT_07s0005g01710)* | CGCTTGCTGACTAGTACTGATA | AAAGGAAAAATACCAAGGTGCC |
| *VvPYR (VIT_02s0012g01270)* | GATTGGAGCTTCTAGATGACGA | ATGATTCGATCACTAGTGTCCC |
| *VvPDC1* *(VIT_13s0067g00340)* | GCAAGAAGGTGATGTTGTCATTGC | ACTTGAGAGATACCGTAGGTGTTG |
| *VvARO10* *(VIT_03s0038g02120)* | TTGCCATGCAAGACCACTCAA | TGCTGCACCGTCACCTTCAA |
| *VvADH1* *(VIT_18s0001g15410)* | GCTGGTGCCAAGTGTTGTTCT | GCTTCTCTGGTGTCAGCTCTGTT |
| *VvACC1* *(VIT_12s0059g01380)* | AAGGTCGCTCTACAAGCAAGAG | TGAGCCATAGGCAACCTTCAC |
| *VvFAS1* *(VIT_01s0011g06640)* | TGCTAGATTGGCCGGAAAGTACA | TCGATGATTTCCTTGATAGGTTCGG |
| *VvOLE1* *(VIT_14s0066g00700)* | GGTATTGTTCACGACGTATCTGGTT | GCCTTGGTAGCGTCCTTACCT |
| *VvELO1* *(VIT_04s0023g01140)* | GCAGTTCATGCTTGATCTCATTGTC | CAATCCTCACACTGTGGAGTACAAG |
| *VvIAH1* *(VIT_10s0003g00960)* | CCGTACCAACGAGAACTTTGCC | GCCAAGCATCACCACCTTCC |
| *VvFAT* *(VIT_05s0094g00930)* | ATTGCCGCTGATCGCTAAGG | CGCTGACGCCGCTATTGAC |
| *VvACX2* *(VIT_00s0662g00010)* | CAGAAAGGAACAAAAGGAGACG | CTCCATCTCGAACACGATCTAA |
| *VvPCK1* *(VIT_07s0205g00070)* | AGGGCTTATCATTCACTGTTCA | GTAGAGGATGTCATGTAGTGGG |
| *VvMFP2* *(VIT_05s0077g02140)* | CTTGCAAGTAACACTTCGACAA | AAAGAAATGGGCTCCAACAATC |
| *VvEEB1* *(VIT_11s0016g01930)* | AGCCATGAGCCTCTCATCAGG | TGAGCAACCTCTAGCATTAAGGAC |
| *VvATF1* *(VIT_16s0039g00570)* | GGACCGAGTTGGCGGCTAAT | GTGGATCGAAGACCGACCATCA |
| *VvFRK (VIT_01s0011g00240)* | GATCTAAATCTTCCGTTGCCAC | CAAGTTCTTGCTTGGTAACCTC |
| *VvTK (VIT_17s0000g08560)* | GTCGGATCAAATCAGAAAGCAA | CACTCCATCATGTGTCACAATC |
| *VvALS* *(VIT_16s0022g01030)* | TGTCTTTTGCTTACGTTGAAGG | CTATCAATTTCAGCGTCTGTGG |
| *VvTPI (VIT_03s0038g01780)* | AAGACACATGGAATGAACAACG | CAATTCCCAACCCACATGTAAA |
| *VvPFK* *(VIT_14s0108g00540)* | CAAACCCTAAACCTCACGAATC | GTTAGAAAACCAACGGACCAAA |
| *VvLDH (VIT_01s0010g03100)* | AAGGTTCTGGAGAGTCTAGACT | CACCGGGTTCGTTACTATAAGT |
| *VvDNL (VIT_13s0019g04580)* | AATTACAGAGGCTGGCTTTACT | GGAAAACAACAGGATCAAGGTC |
| *VvSUS (VIT_11s0016g00470)* | CAGCAGCAACAGCAATAACTAA | GCAGATGCACACACTTTATTGA |
| *vINV* *(VIT_06s0061g01520)* | CCAGAAAACTTGTAGAAGCACC | GTTGACGCATTCCTTAAGGATC |
| *VvGPDH* *(VIT_14s0219g00280)* | TTGTCAAAGGGTATAGAGGCTG | TGTTTTCCATAGCTACACCAGT |
| *VvPGK* *(VIT_19s0085g00380)* | AATACTTGAAACCTTCGGTTGC | CAATTTTCGATGACACCTTCGA |
| *VvNSE (VIT_17s0000g04540)* | CTCAGAGCCTTTGTGAACTCTA | TGCTTCAGCTATTCTCTTTGGA |
| *VvPDH (VIT_09s0002g07930)* | AATTACAGAGGCTGGCTTTACT | GGAAAACAACAGGATCAAGGTC |
| *VvGI* *(VIT_18s0001g07280)* | GCTCATGTATCTCAGCCTTTTG | CTTTTCACCATGAAAGCTGTGA |
| *VvPK* *(VIT_08s0007g04170)* | GATCTAAATCTTCCGTTGCCAC | CAAGTTCTTGCTTGGTAACCTC |
| *VvHT1* *(VIT_00s0515g00050)* | AGAGTTCTTTTCTTAGTCGGGG | GAACCAGAAAAGCGTACACTTT |
| *VvHXK* *(VIT_18s0001g14230)* | GCTGAAAGCTTAATTCCTGGTT | TTCTTGGGCCATCTTTAGTAGG |
