# Supplementary material for: Exogenous Abscisic Acid Mediates Berry Quality Improvement by Altered Endogenous Plant Hormones Level in “Ruiduhongyu” Grapevine
Source: Front Plant Sci. 2021 Oct 1;12:739964. doi: 10.3389/fpls.2021.739964 (PMC8519001; doi:10.3389/fpls.2021.739964)
Supplement: Supplementary Table 2 — Detection and quantification limits of target compounds by HPLC. [file Table_2.docx]

| **Phytohormones** | **Linear range** | **Calibration slopes** | **Correlation coefficient**  **(R^2^)** | **LOD**  **(ng/mL)** | **LOQ**  **(ng/mL)** |
| --- | --- | --- | --- | --- | --- |
| ABA | 2-10000 | 5352.4 | 1 | 1 | 3.33 |
| IAA | 10-200 | 13153 | 0.9999 | 0.71 | 2.34 |
| IBA | 12.5-200 | 9189 | 0.9999 | 2 | 6.62 |
| IPA | 5-200 | 27131 | 0.9999 | 1.09 | 3.59 |
| GA_3_ | 2-32 | 62385 | 0.9997 | 0.96 | 3.17 |
| SA | 1.25-20 | 83943 | 0.9975 | 2.075 | 6.84 |
| JA | 4-5000 | 17.424 | 0.9995 | 0.38 | 1.25 |
| MeJA | 2.5-50 | 23484 | 0.9995 | 0.55 | 1.81 |
| KT | 1-10 | 225488 | 0.9989 | 0.59 | 1.94 |
| ZT | 1.25-12.5 | 1E+06 | 0.9977 | 0.8 | 2.64 |
| ZR | 2.5-100 | 76111 | 0.9998 | 0.73 | 2.41 |
| ip | 0.25-40 | 2E+06 | 0.9999 | 1.04 | 3.43 |
| ipR | 2.5-20 | 14889 | 0.9996 | 0.28 | 0.92 |
